# Glioma: experimental models and reality

**DOI:** 10.1007/s00401-017-1671-4

**Published:** 2017-01-10

**Authors:** Krissie Lenting, Roel Verhaak, Mark ter Laan, Pieter Wesseling, William Leenders

**Affiliations:** 1 Department of Pathology, Radboud University Medical Center, PO Box 9101, 6500 HB Nijmegen, The Netherlands; 2 The Jackson Laboratory for Genomic Medicine, Farmington, CT USA; 3 Department of Neurosurgery, Radboud University Medical Center, Nijmegen, The Netherlands; 4 Department of Pathology, VU University Medical Center, Amsterdam, The Netherlands; 5 Department of Pathology, Princess Máxima Center for Pediatric Oncology and University Medical Center Utrecht, Utrecht, The Netherlands

## Abstract

In theory, in vitro and in vivo models for human gliomas have great potential to not only enhance our understanding of glioma biology, but also to facilitate the development of novel treatment strategies for these tumors. For reliable prediction and validation of the effects of different therapeutic modalities, however, glioma models need to comply with specific and more strict demands than other models of cancer, and these demands are directly related to the combination of genetic aberrations and the specific brain micro-environment gliomas grow in. This review starts with a brief introduction on the pathological and molecular characteristics of gliomas, followed by an overview of the models that have been used in the last decades in glioma research. Next, we will discuss how these models may play a role in better understanding glioma development and especially in how they can aid in the design and optimization of novel therapies. The strengths and weaknesses of the different models will be discussed in light of genotypic, phenotypic and metabolic characteristics of human gliomas. The last part of this review provides some examples of how therapy experiments using glioma models can lead to deceptive results when such characteristics are not properly taken into account.

## Introduction

### Clinicopathology of gliomas

Glioma is a rare cancer (6 diagnoses per 100,000 people annually) of the central nervous system (CNS) originating from (precursors of) glial cells [41]. The vast majority is characterized by diffuse infiltrative growth into the surrounding CNS parenchyma [29]. Based on the astrocytic, respectively, oligodendroglial phenotype of the cancer cells these diffuse gliomas are traditionally histopathologically typed as diffuse astrocytomas, oligodendrogliomas, or as mixed gliomas/oligoastrocytomas. However, the latter diagnosis is disappearing because nowadays molecular testing generally provides an unambiguous diagnosis of either astrocytic or oligodendroglial tumor (see “Molecular pathology of gliomas” and [91]). Furthermore, based on the presence/absence of marked mitotic activity, necrosis and florid microvascular proliferation (MVP) a malignancy grade is assigned to diffuse gliomas (World Health Organization (WHO) grade II–IV) [68, 159].

A shared characteristic of all grades of diffuse glioma is their extensive infiltration in the CNS parenchyma along white matter tracts and pre-existing blood vessels [29]. Surgical cure for diffuse gliomas is not available because complete resection cannot be achieved. Without treatment, survival of patients with glioblastoma (not only the most malignant, but also by far the most frequent glioma) is approximately 6–9 months after diagnosis, and even with current standard treatment using a combination of surgery irradiation and the DNA-alkylating agent temozolomide, survival rates remain dramatically poor [137, 138]. The limited progress in implementing novel, more effective treatment protocols for diffuse glioma sharply contrasts the situation of many other cancer types.

The most frequent examples of gliomas with a circumscribed rather than diffuse infiltrative growth pattern are pilocytic astrocytoma (relatively common in children and generally benign/WHO grade I) and different variants of ependymoma (occurring in both children and adults and with variable aggressiveness corresponding to WHO grade I–III) [33, 53, 153].

### Molecular pathology of gliomas

Especially during the last decade, multiple studies have shed light on the molecular events underlying gliomagenesis and on their clinical relevance as diagnostic, prognostic and/or predictive markers. Some of these markers are now incorporated in the definitions of particular glioma entities in the recently published, revised WHO classification [90, 91]. Heterozygous mutations in the gene encoding the metabolic enzyme isocitrate dehydrogenase (IDH) are seen in 70–80% of grade II and III diffuse gliomas and in 12% of glioblastomas [108, 163]. These mutations (mostly involving cytosolic *IDH1*, lesser so mitochondrial *IDH2*) have important consequences for the epigenome and cause extensive DNA methylation in IDH-mutant diffuse gliomas (‘glioma-CpG island methylator phenotype’/G-CIMP) [22, 104]. In ‘canonical’ oligodendrogliomas, according to the recently published WHO classification of CNS tumors, an *IDH1* or *IDH2* mutation co-occurs with complete and combined deletion of chromosome arms 1p and 19q [20, 44, 56, 105, 164]. This complete 1p/19q codeletion not only constitutes the genetic hallmark of oligodendrogliomas, but also an important favorable prognostic and predictive marker. Diffuse astrocytic tumors can be IDH mutant or wild type. While most IDH mutant astrocytic tumors are at clinical presentation WHO grade II or III, they often progress to a grade IV lesion (‘secondary glioblastoma’). It was recently shown that IDH1-mutated glioblastomas may experience loss of heterozygosity, resulting in absence of wild-type IDH1 expression [57]. It is not known yet whether and how heterozygosity or homozygosity for *IDH* mutations influences biological behavior. The vast majority of diffuse gliomas presenting as glioblastoma at first diagnosis are IDH wild type (‘primary glioblastomas’) [90, 91, 93, 105].

**Fig. 1:**
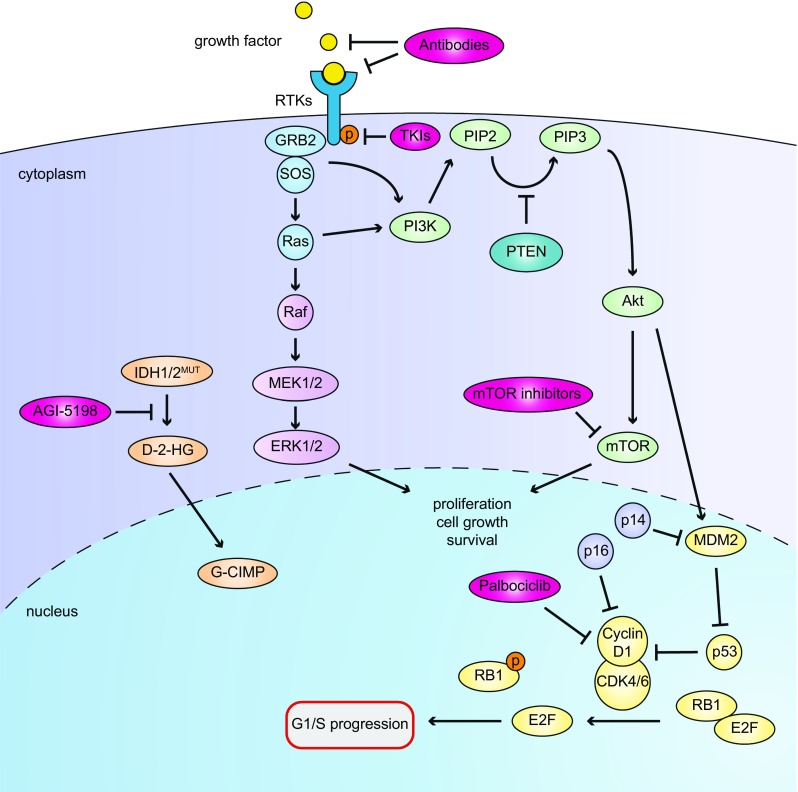
Overview of oncogenic pathways in glioma and possibilities for pharmacological interventions, relevant for the models described in this article. Growth factors bind to RTKs resulting in phosphorylation and Ras/Raf and PI3 K signaling to Akt and mTOR, thus driving cell growth and survival. In a substantial percentage of glioblastomas this process is amplified by loss of the counteracting phosphatase PTEN. Akt also can phosphorylate MDM2 thereby stabilizing the protein. This stabilization causes ubiquitinylation and degradation of the tumor suppressor P53, unleashing cyclin D1 complex activity and leading to uncontrolled G1-S progression. Loss of control over the cell cycle is also induced by *CDKN2A* mutations as CDKN2A products, p14^ARF^ an p16^INK4A^ are important G1-S checkpoint proteins. The PI3 K pathway (activated by phosphorylated growth factor receptor tyrosine kinases [RTKs] such as EGFR, MET or PDGR) may be inhibited by small molecule RTK inhibitors or antibodies directed against ligand binding domains of RTKs or the ligands themselves, thus prevent ligand-receptor interaction. The cyclin D1 pathway (resulting in cell cycle progression from G1 to S phase) may be inhibited by the CDK4/6 inhibitor palbociclib. Finally, mutations in *IDH1* or *IDH2* result in mutant proteins that catalyze the conversion of α-KG to D-2-HG, causing the G-CIMP phenotype and a transcriptional profile leading to gliomagenesis. AGI-5198 specifically inhibits IDH1^R132H^ activity by binding to the catalytic site of the protein

A number of dysregulated pathways are frequently operational in diffuse gliomas that may be amenable for pharmacologic intervention (see Fig. 1). Besides frequent mutations in the promoter of *TERT* (telomerase reverse transcriptase, involved in maintaining telomere length) [44], like in other cancer types three main pathways are frequently (alone or in combination) affected in diffuse gliomas/glioblastomas [19]: (I) the *phosphoinositide 3*-*kinase (PI3* *K)/AKT pathway* is often hyperactive as a result of activating mutations in or amplifications of genes encoding the upstream receptor tyrosine kinases (RTKs) and/or loss of PTEN, a negative regulator of the AKT pathway; (II) *cell cycle control pathways*, e.g. caused by inactivating mutations in *CDKN2A*, the gene encoding the MDM2 inhibitor p14^ARF^ and the cyclin D1-inhibitor p16^INK4A^, or activating mutations in cyclin-dependent kinase (*CDK4*) resulting in uncontrolled progression from the G1 to the S-phase of the cell cycle [20, 150]; (III) *inactivating mutations in TP53*, prohibiting apoptosis of cells with damaged DNA, and resulting in uncontrolled progression of the cell cycle, contributing to the gradual accumulation of mutations and increased intratumoral heterogeneity [134].

Of the most frequent non-diffuse gliomas, pilocytic astrocytomas are almost always affected by single abnormalities of the mitogen-activating protein kinase (MAPK) pathway (most frequently by KIAA1549-BRAF fusion, in other cases by a BRAF V600E or other mutation affecting this pathway), indicating that this neoplasm may be a ‘one pathway’ disease [33]. Ependymal tumors form a very heterogeneous group with regard to not only location, histology and clinical behavior, but also molecular characteristics. Based on detailed molecular analyses combined with clinicopathological information, nine larger subgroups of ependymoma have been identified, some of these carrying a more grim prognosis while others behaving relatively indolent [24, 107].

### Intratumoral heterogeneity

Intratumoral heterogeneity was firmly established by genetic analysis of multiple biopsies of the same tumor, and even on the level of single cells by single-cell RNA-seq analysis [74, 109, 133, 142]. Gliomas may contain subpopulations of cells carrying mutually exclusive amplifications of oncogenes *EGFR* and *PDGFRα* [130]. In line with these observations, recurrent gliomas may be genetically markedly distinct from the tumors from which they originate [71].

A number of the frequently encountered molecular aberrations in diffuse gliomas are targetable with available drugs, examples being inhibitors of RTKs (antibodies and small molecules against among others EGFR, MET, PDGFR [112]), inhibitors of CDK4/6 activity (palbociclib [126]) and inhibitors of mutant IDH enzymes [124] (see Fig. 1). Despite the wealth of information on actionable molecular aberrations and the availability of corresponding targeted drugs, apart from bevacizumab (see “Angiogenesis inhibition in preclinical glioma models”) there has not been any change in approved drug-based treatment strategy for these cancers since the introduction of temozolomide. An important reason for this frustrating notion is the high diversity in genetic aberrations in glioma, combined with substantial intratumoral heterogeneity and the relatively low incidence of diffuse glioma, precluding testing of novel targeted therapies in groups of patients of sufficient size. Furthermore, validated predictive biomarkers for novel targeted drugs are often lacking. It is thus of great importance to have available appropriate preclinical glioma models.

## Preclinical glioma models

Ideally, a preclinical glioma model meets the following requirements; I) Genetic background resembles that of (a subset of) human gliomas; II) Genetic, epigenetic and phenotypic intratumoral heterogeneity is similar to human glioma; III) Model involves an adequate microenvironment with regard to immunocompetence, presence of blood–brain barrier (BBB) and cell-cell interactions (both between tumor cells and with non-neoplastic cells) [106]; IV) Model is reproducible and stable over time.

**Fig. 2:**
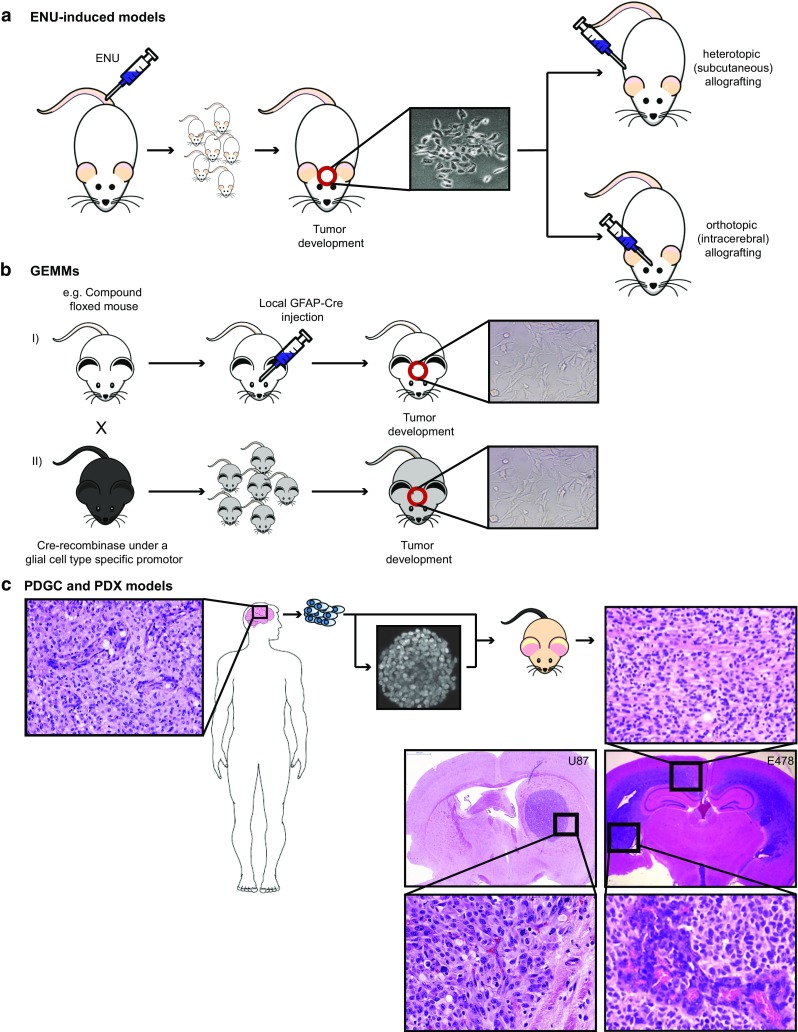
Schematic overview of in vitro and in vivo glioma models. **a** Intravenous injection of ENU into pregnant rat leads to offspring with a high chance of spontaneous glioma development. Resulting gliomas often carry mutations in oncogenes or tumor suppressor genes that are frequently encountered in human gliomas. ENU-induced gliomas have been processed to murine glioma cell lines that can be implanted as allografts in syngeneic animals. **b** GEMMs. Shown are examples of compound flox-ed mouse, in which the floxed modifications can be activated by local transduction of cells with lentiviruses encoding Cre recombinase under control of ubiquitous or cell-type specific promoters, or by crossing with transgenic mice expressing Cre under control of neural- or glial-cell type-specific promoters (e.g., nestin or GFAP promoter). In these cases modifications occur early during embryonal development, unless promoter activity is made inducible. These glioma models are molecularly highly defined, and can be processed to novel murine cell lines that are amenable for allografting. **c** PDGCs or PDX can be generated by preparing cell cultures of surgically obtained glioma material (nowadays mostly spheroid cultures) that can be implanted heterotopically (generally subcutaneously in the flank) or orthotopically (in the brain) of immunocompromised animals; alternatively, surgically obtained human glioma tissue can be directly implanted; ideally, an orthotopic xenograft of a diffuse high grade glioma/glioblastoma in the murine brain recapitulates not only the genotype, but also the phenotype of this tumor with e.g., florid microvascular proliferation (lower right image) and diffuse infiltrative growth in the white matter (upper right image). Of note, as illustrated by a xenograft derived from U87 cells, not all orthotopic glioma models show diffuse infiltration in brain parenchyma, and such models are less relevant for the study of glioma in the context of tumor-brain microenvironment interactions

In the following paragraphs we discuss currently employed preclinical glioma models, including their relevance with respect to molecular make-up, intratumoral heterogeneity, tumor microenvironment, stability and their usefulness for testing of novel therapies. Models that will be discussed can be categorized as carcinogen-induced gliomas in animals, in vitro glioma cell cultures derived from human or animal gliomas, glioma xenograft models (subcutaneous, orthotopic), and transgenic mouse models (Fig. 2). Furthermore, some more ‘exotic’ glioma models (e.g., in zebrafish or fruit flies) will be briefly discussed.

### Murine models of glioma

#### Ethyl-nitrosourea (ENU)-induced gliomas

A widely used model of diffuse glioma that was introduced in the 1970s is based on carcinogen-induced gliomagenesis (Fig. 2a). In this model, pregnant animals (mostly rat) are injected intravenously with a single dose of N-ethyl-nitrosourea (ENU) [127]. The in utero exposure of embryos to this DNA-damaging compound induces predominantly brain tumors, possibly because DNA-repair mechanisms are less active in the brain than in other tissues, resulting in higher rates of stable mutations in neural cells. Interestingly, injection of ENU in adult animals does not result in brain tumors [129], suggesting that neural precursor cells in the developing brain pass through ENU-induced mutations to their progeny, that will cause problems in the event they affect oncogenes or tumor suppressor genes. This model thus may well represent gliomagenesis in humans and allows for the identification of key driver mutations and their roles in glioma development in a spatial and temporal fashion, especially since the tumors in this model have a relatively long latency time (i.e., they become symptomatic only months after birth) [17]. Like in a subset of human gliomas, *TP53* mutations have been identified as key cancer drivers in ENU-induced gliomas, resulting in genetic instability and accumulation of other mutations in oncogenes or tumor suppressor genes [98]. Additionally, other mutations that are frequently encountered in human gliomas, such as amplification of platelet-derived growth factor receptor alpha (*PDGFRα*), deletion of the cell cycle regulator gene *CDKN2A,* and amplification of the EGFR gene can be present in these animal tumors [168], the combination of *TP53* and *PDGFRα* mutations being relatively frequent in pediatric high grade glioma [78, 111, 116]. A recent study demonstrated that a Braf codon 545 mutation (V545E, corresponding to the human BRAF V600E mutation) is a frequent early event in the development of ENU-induced rat gliomas [156]. This mutation can occur in human diffuse gliomas but is more frequently seen in ‘non-diffuse’ gliomas.

The ENU-induced model of gliomagenesis gives rise to genetically heterogeneous tumors and also involves a proper brain microenvironment, including an intact immune system and a blood brain barrier (BBB) [119], making it to a relevant model. One of the downsides of the model is the poorly reproducible character of glioma formation. Consequently, experiments with this model require costly and time-consuming studies with high numbers of animals. Yet, whereas in the early days in vivo visualization of ENU-induced gliomas was impossible, current imaging techniques allow for therapy studies during which animals are longitudinally monitored. Obviously, testing of targeted drugs in this model requires that these are equipotent against the human and murine targets.

#### Transgenic mouse models

The knowledge of the driver mutations that are involved in gliomagenesis has resulted in innovative genetically engineered mouse models (GEMMs) of glioma (for systematic review of GEMMs, see [59]). An elegant approach made use of transgenic animals with GFAP-promoter-driven expression of tv-a, resulting in astrocyte specific expression of this retrovirus receptor. This makes these cells susceptible to infection with avian leukosis virus-derived RCAS vectors that carry expression cassettes for e.g., auto-active EGFR variants [63]. Infection with such viruses will result in astrocytic EGFR hyperactivity. To allow for astrocytic infection, RCAS-vector producing chicken fibroblasts need to be intracerebrally injected, resulting in infection of cells neigbouring the needle track.

Another example in this category is the model created by transcranially injecting lentiviruses expressing Cre-recombinase under control of the glial cell-specific GFAP promoter or the ubiquitously active CMV promoter, in LoxP-transgenic mice, conditionally lacking *p53* or *pten*, and *p16* ^*INK4a*^, and overexpressing the constitutively active KRAS^V12^ mutant (Fig. 2b). In this model high-grade gliomas develop within weeks after Cre-administration that resemble human glioma with respect to phenotype and BBB [38]. In a recent publication Bardella et al. described a transgenic conditional mouse model in which mice carrying a floxed *Idh1* minigene, followed by an *Idh1* ^*R132H*^ allele were crossed with mice carrying a tamoxifen-inducible P^nestin^-Cre transgene [7]. Upon administering tamoxifen to the offspring mice, Cre is selectively expressed in nestin-positive neural progenitor cells in the subventricular zone (see also “Human glioma cell lines: neurosphere cultures”), resulting in deletion of the minigene and activation of the *Idh1* ^*R132H*^ allele in this alleged stem cell population. The resulting cells were more proliferative and displayed invasive behavior, suggestive of an early gliomagenesis phenotype. Such models may well be further developed into lower grade diffuse glioma models.

GEMMs are very suitable to investigate behavior of genetically defined gliomas in an immune competent setting and allow for studies on drug distribution to glioma cells in the brain, taking potential BBB restrictions into consideration [88, 89]. These models however lack the intratumor heterogeneity that is observed in human gliomas. Furthermore, in these models targeted drugs that are tested ideally must have similar activity against human and murine targets in order to predict therapeutic outcome in patients.

#### Murine glioma cell lines and allograft models

The unpredictable character of glioma formation in ENU-induced models has stimulated researchers to create stable in vitro cell line cultures from ENU-induced rat gliomas, among which C6, 9L, T9, RG2, F98 and BT4C and RT-2 (molecular characteristics reviewed in detail in [8]), but also from transgenic mice [132]. The GL261 mouse cell line was generated by intracranial injection of the alkylating agent 3-methylcholantrene into C57BL/6 mice [5, 140]. These murine glioma cell lines have the advantage that they can be implanted orthotopically in syngeneic, immunocompetent animals [8] allowing for the study of tumor immunological aspects. An exception is the C6 glioma model that was generated from an ENU-induced glioma in an outbred strain of Wistar rats. As a consequence, inoculation of C6 cells in common Wistar rat strains results in an allogenic immune response and lack of tumor growth [10]. However, the cell lines that do grow in their syngenic hosts after intracerebral transplantation, develop into invasive cancers that have been used to investigate effects of targeted therapy and radiotherapy [28].

Novel immunotherapy concepts have been introduced in cancer treatment, such as dendritic cell vaccination and immune checkpoint inhibition (reviewed in [146]). Gliomas are considered by some to be good candidates for dendritic cell (DC) vaccination [85], but the efficacy of this and of other immunotherapeutic approaches is currently hampered by the immune suppressive milieu in (patients with) glioma [51, 54]. Glioma allografts in immunocompetent animals are highly valuable models to optimize immune-modulatory therapies and improve immunotherapeutic protocols [166]. Yet, as indicated above, one has to bear in mind that the intratumoral heterogeneity of human gliomas is probably not fully recapitulated in animal models. Another drawback of these murine glioma models in the context of immunotherapy is that humanized antibodies for clinical use are immunogenic in such models, precluding repeated administration. This problem can partly be circumvented by using immune deficient animals (rats or mice), obviously not an adequate solution for studies in the field of immune therapy. Alternatively, immune-humanized mice may be used in which the mouse Ig-locus is exchanged for the human Ig-locus [13, 97, 167]. Clearly, in such models the targeting antibody should be reactive against the glioma target of interest. Genetic engineering of rat glioma cell lines aiming for expression of the human antigen of interest may be helpful in this respect. An example of this latter approach is the evaluation of therapeutic efficacy of anti-EGFRvIII antibodies using allografts of F98 rat glioma cells overexpressing human EGFRvIII [165]. So far, spontaneous *IDH1* mutations have not been reported in murine glioma cell lines, making these models less relevant for the study of IDH mutant human gliomas. Attempts to generate stable cell cultures from transgenic mice, expressing IDH1^R132H^ in nestin-positive neural progenitor cells have so far also failed [7].

### Models of human glioma

#### Human glioma cell lines: conventional cell cultures

In an attempt to work with glioma models that resemble as closely as possible the genetic make-up of their human counterparts, many research groups exploit patient-derived glioma cell lines. The two most widely studied cell lines, U87 and U251, were generated in the sixties of the last century from patients with a glioblastoma [114, 160]. In the past 4 decades experiments with these lines have been reported in over 2000 and 1000 publications, respectively. The U87 genome has recently been fully sequenced [31], and this effort revealed an enormous number of indels, copy number variations and translocations, most of which were probably acquired during decades of cell culture. Indeed, only recently the consequences of cell culture with fetal bovine serum for genetic instability have been recognized [2, 46, 67, 144]. Still, genetic aberrations from the original tumors that are retained in these cell lines allow for the detailed study of their contribution to oncogenic cell signaling pathways in a controlled and reproducible fashion. Furthermore, such cell lines also allow for rapid and reproducible testing of targeted drugs in vitro, as a prescreen for further testing in appropriate preclinical in vivo models.

Of note, U87 cells have found their way to many labs in the world, and it may be expected that, due to genetic drift under serum culture conditions, there is a large number of subclones of U87 available that may affect experimental reproducibility. The need for regular cell line authentication is now widely recognized and cell line authentication is already required by a number of scientific journals [48].

#### Human glioma cell lines: neurosphere cultures

The genetic drift that is caused by culturing cells under serum conditions has resulted in a search for alternatives. In 1992, Weis and Reynolds reported that neural stem cells (NSC) can be stably maintained and propagated as neurospheres when cultured in growth-factor defined media in the absence of serum [121]. A subset of surgically obtained gliomas can be processed to genetically stable cell lines by growing tumor spheroids in a similar specialized medium containing basic fibroblast growth factor (bFGF), epidermal growth factor (EGF) and neuronal viability supplement B27 [6, 16, 34]. Success rates of generating neurosphere cultures from gliomas is however largely dependent on tumor grade and *IDH* status. Especially generation of cell cultures from *IDH*-mutated lower grade (WHO grade II and III) gliomas is difficult, with only few examples reported in the literature [76, 92, 124]. Furthermore, considering the heterogeneous composition of human gliomas, it is quite likely that each patient-derived glioma spheroid line represents only a subset of the most aggressive cells from the original tumor.

This directly leads to the questions how the initiating glioma cells diverge into the heterogeneous population of progeny cells and what exactly are the initiating cells. This issue is still not resolved. Current ideas are that many gliomas originate from neural progenitor cells in e.g., the subventricular zone that during normal development differentiate into neuronal and glial cells. Based on the concepts of stem cells in healthy tissues, these glioma stem-like cells (GSLCs), also called glioma-initiating cells (GICs), are believed to divide asymmetrically, yielding a novel stem-like cell that is resistant to chemotherapy and radiotherapy and is responsible for tumor progression, and a non-stem-like daughter cell [136]. This hypothesis would qualify GSLCs as the cells that are most essential, but also most difficult to eradicate in glioma therapies.

Interestingly, glioma spheroid cultures contain characteristics of GSLCs, and may express stem cell markers such as CD133, Nestin, Sox2 and SSEA-1 [131]. However, expression of these markers varies widely, making it difficult to unequivocally define the initiating cells in gliomas. Furthermore, lack of expression of GSLC markers does not necessarily imply lack of the ability to generate tumors [64]. Even though in vitro and in vivo glioma models are promising tools for further elucidation of the molecular and functional characteristics of GSLCs/GICs (including identification of the best markers for recognizing these cells) [157], so far this has not led to broad consensus on what exactly GSLCs/GICs are and what their impact is in the pathobiology of gliomas.

**Fig. 3:**
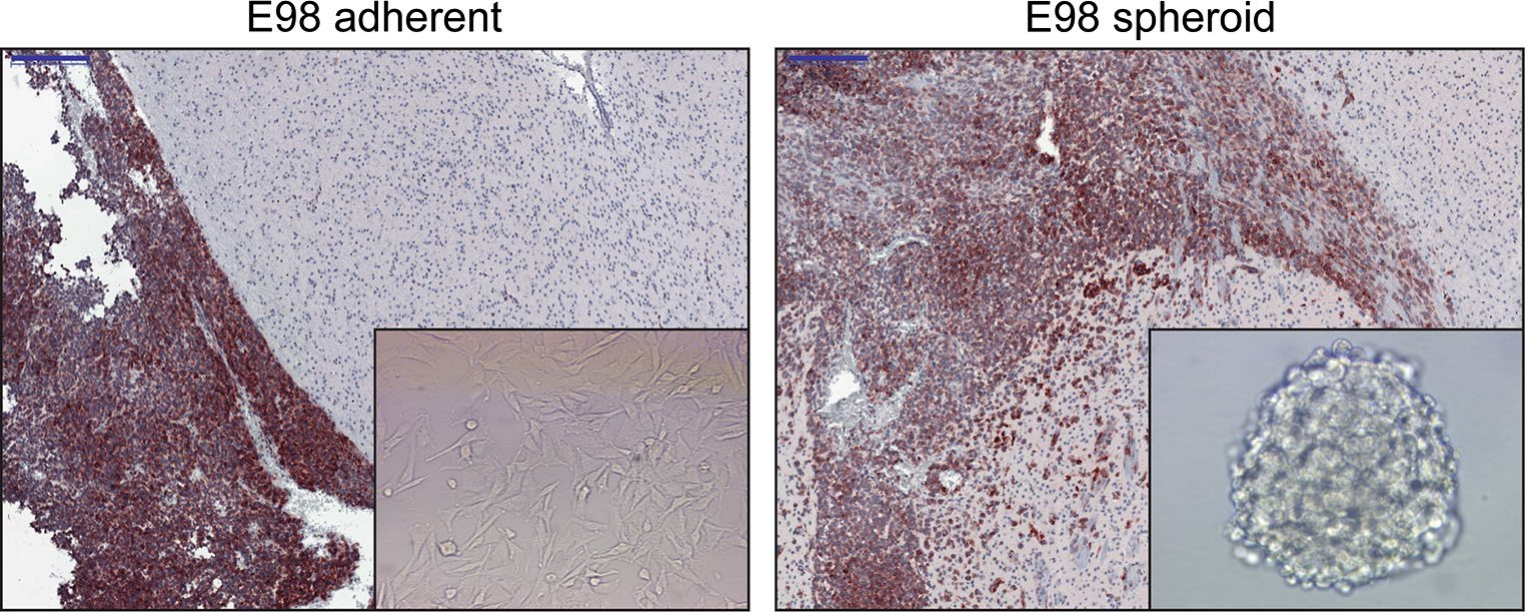
Cell culture conditions impact the phenotype of orthotopic E98 glioma xenografts. E98 glioma cells grown as adherent cells in serum-supplemented culture medium lose the capacity to grow diffusely in the brain (*left panel*). Glioma cells are visualized via immunohistochemical staining for c-MET. Note the sharp demarcation between tumor (growing in the leptomeninges here) and brain parenchyma in the left panel, whereas E98 tumor cells diffusely infiltrate in the brain parenchyma after passaging as neurospheres (*right panel*). Of note, photographs are representative examples from experiments in which E98 cells, grown as adherent cultures or spheroid cultures, were injected in groups of 5 mice using exactly the same injection procedure. Bars indicate 200 µm

When bFGF and EGF in the cell culture medium are replaced with serum, cells rapidly lose intercellular connections within the neurospheres and undergo marked phenotypical changes: cells start to adhere to plastic, flatten, acquire a more fibroblast appearance, and loose GSLC markers [82, 110]. When testing the effects of different culture conditions using the E98-model (derived from a human glioblastoma [30]) in our lab, upon intracerebral implantation only E98 cells cultured as neurospheres retained the capacity to diffusely infiltrate into the brain parenchyma, whereas E98 cells grown under serum conditions had lost this capacity (Fig. 3). Similar observations were made by others [82]. The exact mechanism underlying this phenomenon is unclear, but may have to do with a GSLC population with invasive potential that is enriched in tumor spheroids [135, 139].

Nowadays, glioma cultures can be relatively easily modified with CRISPR/Cas9 technology, allowing for the elucidation of the role of individual oncogenes and tumor suppressor genes. Furthermore, it is now common practice to generate luciferase-expressing glioma cells, enabling longitudinal non-invasive follow up of tumor development in the mouse brain [12, 21].

#### Heterotopic versus orthotopic grafts of glioma cell lines

Subcutaneous xenografting of human glioma cells in immunocompromised mice is a frequently used approach to obtain preclinical proof of concept for the efficacy of targeted drugs [123]. Glioma xenografts grown in the subcutaneous space have the advantage that tumor development can be followed visually and quantified in terms of tumor dimensions, allowing rapid testing of treatment efficacy. Such experiments may yield valuable results, especially if the drugs directly act on tumor cells (e.g., inhibitors of hyperactive oncogenes). Caution is however in place when drug activity is based on disturbing the interaction with the tumor microenvironment, such as in case of anti-angiogenic drugs or particular metabolic inhibitors. Subcutaneous glioma models lack the appropriate CNS microenvironment that has an essential role in glioma biology. Studies with such subcutaneous models may result in overinterpretation of effects of angiogenesis inhibitors [155] (see “Angiogenesis inhibition in preclinical glioma models”).

#### Orthotopic patient-derived xenografts

The desire to circumvent culture-related problems as described above combined with the increased understanding that glioma biology and therapy are heavily influenced by interactions between cancer cells and their microenvironment have increased the interest in the use of orthotopic glioma xenografts. Freshly obtained surgical glioma samples can be directly injected into the brains of immune-deficient mice using stereotactic devices or by a freehand procedure and maintained by serial transplantation [30, 76, 92]. Using such an approach we have created the *IDH1* ^*R132H*^-E478 xenograft model that is genetically highly similar to the anaplastic oligodendroglioma from which it was derived [102]. RNAseq analyses of such models allows the discrimination of human and mouse transcripts, preventing contaminating contributions of host non-neoplastic cell transcripts (which is an unavoidable flaw of studies on clinical cancer specimens).

A drawback of such glioma models is that xenografting is performed in immune-deficient animals and there will be a selection for the fastest growing cell clones resulting in reduced intratumor heterogeneity. Furthermore, orthotopic implantation of cell lines is not a guarantee for a clinically relevant phenotype: whereas orthotopic U251 xenografts have a striking phenotypic resemblance to human GBM [96], including diffuse infiltration in the brain parenchyma and palisading necrosis, U87 cells generally develop to bulky, sharply demarcated lesions that lack such infiltrative growth [29, 69] (see also Fig. 2).

In light of the highly promising results that are obtained with immune checkpoint inhibitors for other cancer types, reconstituting a human immune system in xenografted mice will be of high importance to test these therapies for glioma in a preclinical setting [97, 167]. Whether patient-derived xenograft (PDX) models can be optimized by using humanized mice to allow for testing of clinically available immune checkpoint inhibitors, remains to be seen.

### Other glioma models

The vast majority of glioma models reported in the literature concerns models for diffuse gliomas, but preclinical models for non-diffuse gliomas such as pilocytic astrocytomas and ependymomas have been reported as well (in vitro, heterotopic or orthotopic murine models, GEMMs) [55, 72, 95, 115]. Obviously, like for diffuse gliomas, in an ideal situation the preclinical models for such gliomas are reproducible and closely resemble their human counterparts with regard to genetic background, intratumoral heterogeneity and tumor microenvironment.

As generation of mouse and rat models of glioma can be a slow process, in vivo detection of orthotopic gliomas requires sophisticated equipment, and drug screenings in these models are time-consuming and costly endeavours, some more ‘exotic’ glioma models have been developed. A potentially interesting example is the zebrafish (*Danio rerio*) model for the study of gliomas [154, 158]. Zebrafish embryos are increasingly used for cancer studies since the discovery that pathways of tumorigenesis are similar in humans and zebrafish [66]. In a typical experiment hundreds of day 3 post-fertilization zebrafish embryos are injected with tumor cells, stained with a fluorescent membrane dye. Using the pigmentation-mutated casper strain of zebrafish that remain transparent during life, cancer cells can be readily visualized using UV microscopy. Intracerebral implantation of human glioma cells in zebrafish was shown to result in xenografts with phenotypes that were similar to xenografts in mouse, grown from the same cells [45]. The zebrafish system allows semi-high-throughput drug screening, e.g., by adding compounds in the water [158]. A potentially serious drawback of the zebrafish system is that glioma cells need to adapt to function at 32 °C, with possible consequences for metabolism and activity of oncogenic pathways. Furthermore, one needs to take into account that in zebrafish the BBB starts to develop from day 3 post fertilization and is fully developed only at day 15 post fertilization, and that the immune system has not matured in the early stages of development as well [80, 162]. Furthermore, it is not yet clear whether the zebrafish accommodates growth of all sorts of glioma cells, including the difficult-to-grow *IDH* mutant gliomas.

Another model of interest is the fruit fly (*Drosophila melanogaster*), a highly versatile genetic model system in which specific gene functions can be manipulated in a single-cell fashion in an in vivo setting [161]. This provides the opportunity to investigate the effect of genetic aberrations in an intact nervous system. Many molecular pathways, such as the RTK signaling pathways, are highly conserved between invertebrates and humans [120]. It has been reported that these models may recapitulate key characteristics of glioblastoma with regard to increased proliferation and migration [117].

The downsides of available experimental animal models of glioma has raised interest in using dogs with naturally occurring gliomas for testing of novel drugs and optimizing novel treatment concepts [81]. Although canine gliomas probably represent a good intermediate between murine models and humans, being more relevant in terms of intratumoral heterogeneity and immune system, low incidence and lack of canine-specific molecular testing facilities, hamper larger studies.

A recent development has been that human fibroblasts after p53 knock down are converted to inducible pluripotent stem cells (iPSCs) that can subsequently be differentiated to neural progenitor cells to yield TP53-mutated NPCs. These cells can be transformed to glioma initiating cells by lentiviral transduction of oncogenes [128].

**Table 1:** Summary of available models of glioma and their strengths and weaknesses

Model type	(Epi)genetic make up	Heterogeneity	Immunocompetent	Brain micro-environment	Blood–brain barrier	Stable/reproducible
ENU-induced murine tumors	Partly relevant (among others p53, braf, pdgfrα). No IDH mutations identified	Genetically heterogeneous, different neural cells may be initiator cells	Yes	Relevant	Yes	No
GEMMs (conditional expression of oncogenes/loss of tumor suppressor genes)	Partly relevant, only few known driver mutations used (among others loss of p53/pten,CDKN2A, kras^V12)^ [38] Existing models expressing IDH1-R132H in neural stem cells have epigenetic alterations but are not tumor models [7]	Genetically homogeneous, initiator cell type dependent on promoter driving Cre expression (CMV/nestin/GFAP)	Yes	Relevant when Cre expression is induced in the CNS (intracerebral injection of Cre-lentivirus, crossing mice with developmental CNS- expression of Cre	Yes	Yes
PDX—subcutaneous	Partly relevant	Genetically homogeneous, but intratumoral heterogeneity due to lack of pre-existent vasculature, development of hypoxia and angiogenesis dependence	No	Non-relevant	No	Yes
PDX—orthotopic	Relevant	Partly, it is not known to which extent PDX models represent most aggressive parts of the originating tumor	No	Partly, only relevant in case PDXs have retained capacity to grow via diffuse infiltration	Yes	Yes
Cell lines—adherent	Less relevant	No	No	Non-relevant	No	Yes
Cell lines—spheroids	Possibly relevant	No	No	Non-relevant	No	Yes
Zebrafish	Non-relevant	No	No	Probably non-relevant	No	Yes
Canine	Possibly relevant	Yes	Yes	Relevant	Yes	No
Fruit fly	Not relevant	No	No	Relevant	No	No

## Glioma models and predictive medicine; a reality check

The exact glioma model used is an important determinant for the outcome of preclinical therapeutic studies. After briefly underscoring the importance of the molecular underpinnings and of the microenvironment of the model, anti-angiogenic therapy and tumor-cell metabolism will be discussed in more detail as examples where exploitation of inappropriate models may easily lead to deceptive information.

### The importance of molecular underpinnings and microenvironment

**Fig. 4:**
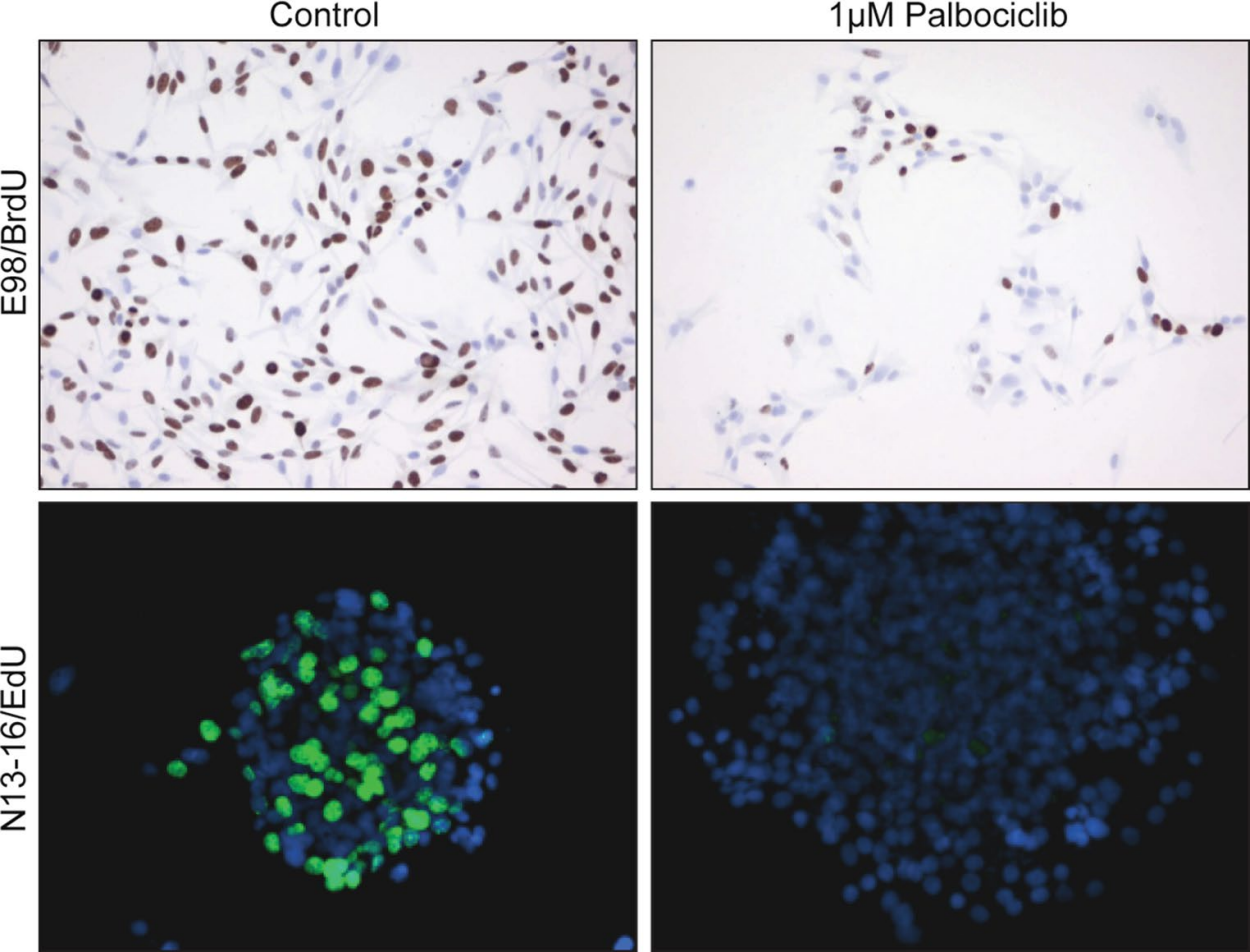
Example of promising in vitro effect of *CDKN2A*-mutation targeting that is difficult to translate into clinical efficacy. Two different patient-derived glioblastoma models (E98 adherent cells, N13-16 spheroid cultures, both characterized by dysfunctional CDKN2A), were incubated with placebo (*left panels*) or with the CDK4/6 inhibitor palbociclib (*right panels*). Visualization of DNA synthesis (S-phase of the cell cycle) with BrdU or EdU (as indicated) shows that palbociclib effectively prevents entry into the S-phase. Although such in vitro results are very promising, also because 80% of human glioblastomas have dysfunctional CDKN2A leading to a defective G1-arrest, in vivo diffuse gliomas may well be protected from palbociclib because this drug is substrate for p-glycoproteins of the BBB [36]

Lots of (combinations of) mutations that are common in human glioma, are retained in human glioma cell lines. *In vitro* studies using appropriate cell lines are therefore well suited to investigate interactions between aberrant pathways and establish optimal concentrations of inhibitors, e.g. to achieve potential synthetic lethality [75]. A large proportion of gliomas have a dysfunctional *CDKN2A*, the gene encoding the cyclin D1 inhibitor p16^INK4A^. These cells have a defective G1-S checkpoint that can however be corrected pharmacologically by palbociclib. This CDK4/6 inhibitor has recently been approved by the FDA and the European Medicine Agency (EMA) for treatment of women with advanced breast cancer [47]. Treatment of patient-derived p16^INK4a−/−^glioma cell lines, grown as adherent cells or as spheroids, with palbociclib results in an effective block of cell cycle progression already at µmolar concentrations as shown by incorporation of nucleotide analogues bromo-deoxyuridine (BrdU) and 5-ethynyl-2′-deoxyuridine (EdU) (Fig. 4, unpublished work). These results confirm literature data that a large percentage of patient-derived glioma explants respond to palbociclib [23]. However, its clinical usability is strongly restricted because palbociclib is a substrate for P-glycoprotein (a family of ATP-dependent transporter proteins in the BBB that pump out the recognized substrate), reducing drug distribution behind the BBB [36]. Once the BBB limitations are overcome (see below) palbociclib may indeed be used to improve prognosis for a large proportion of glioma patients.

Hyperactivity of the phosphoinositol-3-phosphate (PI3 K) pathway in glioblastoma is common, due to (a combination of) amplified or otherwise mutationally activated oncogenic RTKs, and/or loss of the phosphatase *PTEN* [27, 35]. Aberrant expression of epidermal growth factor receptor (EGFR) and/or the ligand-independent EGFR mutant EGFRvIII (a product of a genetic deletion resulting in loss of exons 2-7 in the mRNA) is a frequent phenomenon in glioblastoma [26, 60], driving growth and migration of tumor cells [141]. Alterations in other RTKs that are frequently encountered in glioblastoma are mutations in *PDGFRα* and *MET* [103]. Also these mutations are retained in cell lines. We previously showed that targeted inhibition of MET by cabozantinib is highly effective in E98 cells in vitro, blocking MET phosphorylation and cell proliferation with IC_50_ in the nmolar range. Testing the same drugs in orthotopic xenografts generated from the same cell line resulted in increased survival of mice, yet did not prevent development of treatment resistant cancers [100]. Such differences in effects of the same drug on the same cell line in vitro and in vivo illustrates the important role of the tumor microenvironment.

The BBB is designed to protect neural tissue from toxic substances in the blood. In large areas of diffuse infiltrative glioma this barrier also shields cancer cells from drugs. The BBB is composed of a relatively impermeable layer of endothelial cells that communicate with astrocytes via contacts with astrocytic end feet [9, 79]. Except for creating a physical barrier, endothelial cells of the BBB express a large diversity of P-glycoprotein family members, making the BBB impermeable to the vast majority of drugs [15, 39, 87, 89, 149]. Therefore it is of high importance that drugs that have been positively tested on cell lines are subsequently tested in appropriate preclinical models that have retained diffuse growth, and in which cancer cells may ‘hide’ behind the BBB. For drugs that cannot pass the BBB, there is the need to find solutions for local and controlled BBB disruption, or to discover novel targeted drugs that are not substrate for the P-glycoprotein family of drug transporters [87, 89, 149]. Numerous research efforts to get drugs over the BBB are ongoing, including active transport of peptide- or antibody-coated nanoparticles [50, 73, 145]. Mechanical temporary and spatially restricted disruption of the BBB with high intensity focused ultrasound (HIFU) [32, 61, 77] or via stereotactic radiotherapy [4] is an alternative approach that may carry promise for the future but needs more preclinical validation.

One of the recent breakthroughs in oncology has been the implementation of immune checkpoint inhibitors, comprising antibodies against programmed death-1 (PD-1, nivolumab) or cytotoxic T-lymphocyte antigen-4 (CTLA-4, ipilimumab) on cytotoxic T-lymphocytes. These antibodies prevent the immune-suppressive interactions between cancer cells and cytotoxic T-cells, boosting anti-tumor immunity [152] and have now been FDA- and EMA-approved or are in clinical trial for a large number of tumor types. With currently available glioma models, preclinical testing of such highly promising approaches is an enormous challenge. PDXs are unfit for this purpose, given the immunocompromised status of the recipient mice, and humanized mice are probably required for preclinical testing of these concepts. Syngeneic models would require that murine antibodies against the murine equivalents of PD-1 and CTLA-4 are generated, assuming that these immune checkpoint systems work alike in humans and rats or mice. Possibly, ENU-induced gliomas would recapitulate human gliomas best with respect to genetic heterogeneity and immunocompetence, but as discussed above this model lacks reproducibility.

### Angiogenesis inhibition in preclinical glioma models

Whereas most targeted therapies in oncology focus on aberrations in tumor cells, anti-angiogenic treatment using bevacizumab, one of the very few FDA-approved new therapies for glioblastoma in the USA, is targeting the interactions between tumor cells and the tumor microenvironment. Based on the VEGF-induced florid MVP that is characteristic for glioblastoma, this cancer type has historically been looked at as angiogenesis-dependent, hence amenable for targeting with VEGF-pathway inhibitors. Obviously, in vitro glioma cell cultures are not suitable to investigate this concept. Lots of studies have concentrated on patient-derived glioma cell lines that were grown as subcutaneous xenografts in immune-deficient mice [11, 86]. These studies generally yielded highly promising results, with tumor stabilization or even regression, whether with anti-VEGF antibodies or with small compound VEGF RTK inhibitors. Combined data from such studies and radiological responses in phase II clinical studies for recurrent glioblastoma resulted in accelerated FDA-approval for bevacizumab as first line treatment for recurrent glioblastoma in 2009 [99]. Unfortunately, large phase III trials testing bevacizumab for newly diagnosed glioblastoma showed no positive effect on overall survival, often despite an initial radiological response [25, 52].

It is now broadly accepted that the FDA approval of anti-VEGF-A treatment for recurrent glioblastoma in 2009 was at least partly based on cases showing a radiological pseudoresponse (resulting from ‘normalization’ of activated tumor blood vessels) rather than from a tumoricidal response [14, 70, 151, 152]. Of note, this phenomenon was demonstrated before in different animal models. For example, VEGF-expressing melanoma metastases in the murine brain that were readily visible in contrast-enhanced MRI became invisible upon treatment with the VEGFR2 inhibitor vandetanib, even while they progressed by growing in the space of Virchow-Robin via vessel co-option [84]. Similarly, several studies using orthotopic models of diffuse glioma had shown that these tumors can grow in CNS tissue independent of angiogenesis, especially cells that overexpress EGFR or MET [103, 141]. Accordingly, anti-angiogenic treatment does not prevent growth of these cancers [101] but it can increase hypoxia and glycolysis in angiogenic parts of glioma [58]. In this situation treatment with angiogenesis inhibitors diminishes contrast-enhancement in MRI scans without preventing diffuse infiltration in the brain parenchyma [100, 101], copying the phenomena seen in the clinic. Meanwhile, reduction of hyperpermeability of brain tumor microvessels by anti-VEGF-A treatment may have a rapid positive effect on edematous brain swelling, thereby temporarily improving the quality of life [37, 70].

**Fig. 5:**
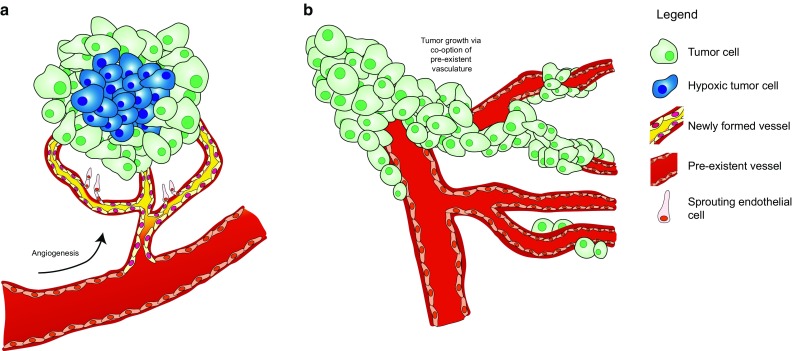
Schematic overview of tumor growth via angiogenesis versus vessel co-option. **a** Schematic representation of the dogma of angiogenesis-dependent growth of a tumor. When a tumor outgrows the capacity of the vasculature, hypoxic stress (indicated by blue cells) initiates sprouting angiogenesis as a rescue pathway (here presented as yellow vessels. **b** Especially in tissue with rich pre-existent vasculature (such as brain tissue), tumors may grow through vessel co-option in an angiogenesis-independent fashion

It is important to consider the cellular principles underlying the different responses of subcutaneous and intracerebral tumors to anti-angiogenic treatment. Tumor growth in the originally avascular, subcutaneous space requires that tumor cells can initiate transcriptional programs that rescue them from starvation [62]. Normally this rescue is accomplished by switching on hypoxia-induced transcriptional programs in cells that are located beyond ~ 100–200 µm from the most nearby blood vessel (the maximum range of oxygen diffusion), including expression of the chemotactic factor VEGF-A, leading to blood vessel growth towards the tumor [125]. The situation in brain, one of the most vascularized organs in the body, is entirely different. Here, diffuse infiltrative glioma cells can incorporate the abundantly present, pre-existent brain microvessels (among others via vessel co-option) [40, 83] (Fig. 5). Of note, orthotopic implantation of glioma cells may not necessarily lead to an adequate phenotype for diffuse glioma. For instance, orthotopic implantation of U87 cells generally results in bulky, well-demarcated tumors with a disrupted BBB, lacking diffuse infiltrative growth along white matter tracts [49]. Anti-angiogenic studies using subcutaneous or ‘bulky’ orthotopic glioma models thus carry the risk of overinterpretation of the therapeutic results, even more so if these studies use drugs that do not pass the BBB.

### Metabolic considerations

**Fig. 6:**
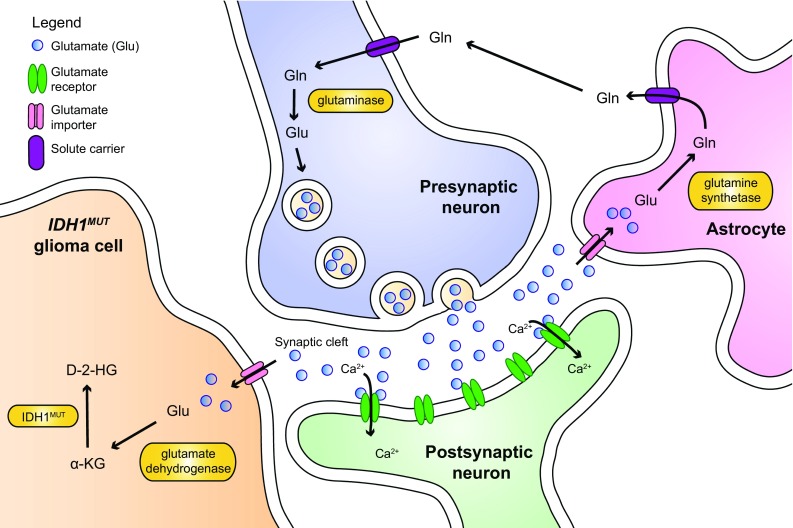
Schematic representation of the glutamine-glutamate cycle in the brain. In the brain, glutamate (Glu) is released by presynaptic neurons to the synaptic cleft. Glutamate activates glutaminergic receptors that undergo a conformational change to allow influx of extracellular calcium. This triggers membrane depolarization in the postsynaptic neuron and induces signal transduction. The excess of glutamate in the synaptic cleft has to be removed to prevent excitotoxicity. Astrocytes take up surplus glutamate through various glutamate importers and convert it to glutamine (Gln) through glutamine synthetase. Glutamine is exported to the capillaries (not shown), or is transferred back to the neurons. Subsequently, neurons can then convert back the glutamine to glutamate, closing the glutamine-glutamate cycle. Glutamate can be imported by *IDH* ^*mut*^ glioma cells in order to supply cells with αKG as a rescue pathway

Another important reason to study glioma in the orthotopic setting relates to tumor cell metabolism [42]. In the brain, ubiquitous amounts of glutamine and the neurotransmitter glutamate are present as part of the glutamine-glutamate cycle (Fig. 6). Together with glucose, glutamine and/or glutamate may be important carbon and nitrogen donors for glioma cells [3], especially those with *IDH* mutations. The biology of *IDH* mutations has been extensively studied and is covered in excellent reviews e.g. [1, 18]. Whereas wild type IDHs produce α-KG and NADPH from isocitrate and NADP^+^, IDH mutants (mostly hotspot mutations IDH1^R132X^ or IDH2^R140X^/IDH2^R172X^) consume α-KG and NADPH while producing the oncometabolite 2-hydroxyglutarate (D-2-HG) [163]. This has two important implications. Firstly, since α-KG and NADPH take important roles in fundamental processes such as fatty acid synthesis and maintenance of redox potential, expression of mutated IDH results in metabolic stress [147, 148]. Secondly, D-2-HG is an inhibitor of a large group of α-KG-dependent enzymes that are involved in epigenetic regulation and induces G-CIMP (see also “Molecular pathology of gliomas”) [43].

We previously suggested that one of the reasons for the unique phenotype of diffuse growth of *IDH*-mutated gliomas relates to the metabolic stress, translating into addiction to glutamate as an alternative carbon source, making this neurotransmitter to a potential chemotactic factor [148] (Fig. 6). Because not all culture media routinely contain this non-essential amino acid, glutamate dependence would be incompatible with standard in vitro growth conditions and could partly explain the difficulty with producing stable glioma cell cultures carrying endogenous IDH mutations [113]. Only in the last years a number of IDH-mutated glioma models have become available, mostly as patient-derived orthotopic xenografts [76, 92, 102, 124].

The high incidence of *IDH*-driver mutations in gliomas, combined with the lack of appropriate in vitro cell cultures has resulted in numerous studies in which effects of IDH-mutants are tested in cell models utilizing overexpression of mutated IDH. Whereas such models have proven valuable to investigate epigenetic phenomena and metabolic fluxes, they may also result in misinterpretation, depending on the specific cell system and desired read-out. For example, *IDH*-wild-type glioma cells routinely express the enzyme branched-chain amino acid transferase-1 (BCAT1) that converts α-KG to glutamate [143]. Together with the antiporter System Xc-, BCAT1 is responsible for maintaining redox potential. Glutamate is secreted from the cell via this transporter in exchange for cystine that entails reductive power [122]. In gliomas carrying the endogenous IDH mutation, *BCAT1* is silenced via promoter hypermethylation [94, 143]. Consequently, glutaminolysis in these cancers is a one-way reaction from glutamine/glutamate to α-KG. Introduction of a mutated IDH gene in a cell with wild-type IDH background will thus result in a competition between BCAT-1 and mutated IDH1 for α-KG, a situation that does not reflect the endogenous situation. Whether full penetrance of D-2-HG-induced epigenetic effects in engineered cell lines can be achieved to the extent that the metabolic phenotype of clinical *IDH*-mutated glioma is fully mimicked, is an open question. Experience so far is that IDH^mut^ introduction in cells results in a gradual loss of the mutated protein, suggestive of a selection against mutant-expression, resulting in selective overgrowth of cells with no or low expression levels of the mutated protein [6, 113]. Introduction of mutant IDH disrupts metabolic balances that may result in model-specific artefacts.

## Summarizing remarks

Glioma models that have been developed so far have greatly enhanced our understanding of glioma formation and metabolism and have contributed to the development and implementation of novel treatment strategies. As a recent example, partly based on in vitro studies and in vivo observations in mice and rabbits, in 2015 the FDA approved Tumor Treating Fields (TTF) as a therapeutic modality for patients with newly-diagnosed glioblastoma (http://www.fda.gov/NewsEvents/Newsroom/PressAnnouncements/ ucm465744.htm) [65].

Optimal exploitation of glioma models remains challenging though. Clearly, diffuse glioma remains a difficult tumor to treat, not in the least because of its diffuse infiltrative growth and the substantial inter- and intratumoral heterogeneity [118]. Up until now, creating adequate models for IDH mutant gliomas proves to be very difficult. Also, intratumoral heterogeneity of diffuse gliomas cannot be recapitulated to the fullest in in vitro or in vivo models. For several therapies the BBB (which is relatively intact in large parts of diffuse glioma) is a true barrier, and modeling this barrier is a difficult task in itself. Yet another challenge lies in the development of appropriate models that can be used to test immunotherapy; in vitro studies using cell lines and xenograft models in immunodeficient animals are inherently unfit for this purpose. So far, a preclinical model that perfectly recapitulates (one or another subtype of) human glioma does not exist. Still, the models that are available may be very useful, but it is of paramount importance to carefully select the most appropriate glioma model for a particular research question, meanwhile realizing not only the strengths but also the weaknesses of the model used.

## List of abbreviations

BBB: Blood brain barrier
CNS: Central nervous system
D-2HG: d-2-hydroxyglutarate
EMA: European Medicine Agency
FDA: Food and drug administration
FDG-PET: Fluorodeoxyglucose-positron emitter tomography
G-CIMP: Glioma CpG island methylator phenotype
GEMM: Genetically engineered mouse model
GIC: Glioma initiating cell
GSLC: Glioma stem-like cell
2-HG: 2-Hydroxyglutarate
HIFU: High intensity focused ultrasound
IDH: Isocitrate dehydrogenase
aKG: Alpha ketoglutarate
MVP: Microvascular proliferation
PDGC: Patient-derived glioma cell
PDGFRα: Platelet-derived growth factor receptor alpha
PDX: Patient-derived xenograft
RTK: Receptor tyrosine kinase
TCA: Tricarboxylic acid cycle
VEGF-A: Vascular endothelial growth factor A
WHO: World Health Organization
